# Thermal separation of soil particles from thermal conductivity measurement under various air pressures

**DOI:** 10.1038/srep40181

**Published:** 2017-01-05

**Authors:** Sen Lu, Tusheng Ren, Yili Lu, Ping Meng, Jinsong Zhang

**Affiliations:** 1 Key Laboratory of Tree Breeding and Cultivation of the State Forestry Administration, Research Institute of Forestry, Beijing 100091, China; 2 Co-Innovation Center for Sustaintable Forestry in Southern China, Nanjing Forestry University, Nanjing 210037, China; 3 Department of Soil and Water, China Agricultural University, Beijing 100193, China

## Abstract

The thermal conductivity of dry soils is related closely to air pressure and the contact areas between solid particles. In this study, the thermal conductivity of two-phase soil systems was determined under reduced and increased air pressures. The thermal separation of soil particles, i.e., the characteristic dimension of the pore space (*d*), was then estimated based on the relationship between soil thermal conductivity and air pressure. Results showed that under both reduced and increased air pressures, *d* estimations were significantly larger than the geometrical mean separation of solid particles (*D*), which suggested that conductive heat transfer through solid particles dominated heat transfer in dry soils. The increased air pressure approach gave *d* values lower than that of the reduced air pressure method. With increasing air pressure, more collisions between gas molecules and solid surface occurred in micro-pores and intra-aggregate pores due to the reduction of mean free path of air molecules. Compared to the reduced air pressure approach, the increased air pressure approach expressed more micro-pore structure attributes in heat transfer. We concluded that measuring thermal conductivity under increased air pressure procedures gave better-quality *d* values, and improved soil micro-pore structure estimation.

Information on the thermal conductivity (*λ*) of a two-phase granular material under various air pressures reflects its intricate pore structure. According to the kinetic theory of gases, numerous collisions occur within gas molecules and among gas molecules and solid particles during heat transfer. The *λ* of a dry soil, which consists of solid and gas phases, generally decreases as air pressure is reduced. This phenomenon is a result of the increase in the mean free path of air molecules (*l*), which is larger than the physical distance between soil particles^1^. Woodside and Messmer^1^ applied the kinetic theory of gases to soil heat transfer and developed a general method to calculate the mean separation of solid particles from *λ* measurements under various air pressures.

The characteristic dimension of the pore space (*d*), which represents the mean separation of solid particles, is important in studying pore structure and specific surface area (*SA*). However, few studies have been performed to determine *d* quantitatively. Momose and Kasubuchi^2^ obtained the *d* values of three soils using λ measurements under reduced pressures. They further defined *d* estimates as thermal separation of soil particles, since the *d* values were obtained from heat transfer experiment. They also applied a static method to estimate the characteristic dimension of the pore space from measured *SA*. Unfortunately, the estimated values from the two methods differed significantly in magnitude. Momose and Kasubuchi^2^ attributed the difference to the discrepancies between the estimation procedures, i.e., results from the heat transfer method represented the dynamic mean separation of particles, whereas the measurements from the *SA* method were the static mean separation of particles from geometry.

Kinetic theory of gases states that a high air pressure (i.e., larger than atmospheric pressure) increases gas density and reduces the mean free path of air molecules. At high air pressures, more collisions occur between gas molecules and the solid surface in micro-pores and intra-aggregate pores during heat transfer in two-phase soils. Understanding heat transfer mechanisms under increased air pressure may reveal soil micro-pore structure. To our knowledge, no information about *d* characteristics under increased air pressure is available. Furthermore, it is unclear whether *d* estimates under increased air pressure differ from that under reduced air pressure.

The objectives of this research are: (1) to study the mean separation of particles under both reduced and increased air pressures; and (2) to compare the dynamic *d* estimates from the heat transfer procedure versus the static *d* results obtained with the *SA* approach.

## Theory

The motion of molecules in gas is complicated because the molecules collide with each other in a continuous random way. The average distance traversed by a molecule between collisions is defined as the mean free path (*l*). It represents the mean distance that the gas molecules travel between one collision and the next collision^3^. According to the kinetic theory of gases, the thermal conductivity of gas (*λ*_g_) is^3^,


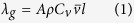


where *A* is a constant, ρ is the density of gas, *C*_*v*_ is the specific heat of gas at constant volume, 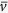 is the mean molecular velocity, and *l* is the mean free path of gas molecules.

Heat transfer by gas molecules in soil pores differs from that in free air. In porous media, the gas enclosure length among particles varies greatly. In certain micro pores, the gas space length is smaller than the mean free path of the gas molecules, and the actual mean free path approaches the linear dimension of the gas enclosure among particles^1^. In these micro pores, therefore, the inverse relation between *l* and gas pressure (*P*) no longer holds, and the thermal conductivity of gas becomes proportional to *P*^1^,^2^.

In the case of a gas enclosed in a porous media, the collisions between solid surface and gas molecules should be taken into account in estimating *λ*_g_. Thus, the effective mean free path of gas 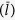 is used to calculate *λ*_g_ instead of *l* in Eq. (1)^1^,^2^


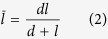


where 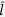 is the effective mean free path of gas in the porous medium, and *d* is the characteristic dimension of the gas space among particles.

Thus, the thermal conductivity of the air enclosed in a porous medium is expressed as^1^,^2^,


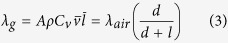


where *λ*_air_ is thermal conductivity of air at the normal pressure.

According to the kinetic theory of gases, *l* of air molecules depends on both temperature and pressure, which is expressed as^4^


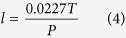


where *l* is in mm, *T* is in Kelvin, and *P* is in Pa.

Substituting Eq. (4) into Eq. (3) gives


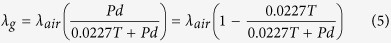


Equation (5) shows that the *λ* of air in a porous medium increases as air pressure becomes larger.

For low *λ*_g_ values, the thermal conductivity of dry granular materials is described with the following equation^1^:


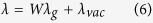


where *λ*_vac_ represents the thermal conductivity of dry granular materials under vacuum, and *W* is a constant that depends on the material types.

A general equation that describes the thermal conductivity of dry granular materials under various pressures is obtained by combining Eq. (5) and Eq. (6),


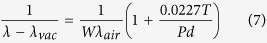


Equation (7) indicates that 1/(*λ* − *λ*_vac_) changes linearly with 1/*P*. Therefore, by using Equation (7), the *d* value of *a* dry granular material could be estimated from *λ* measurements under various pressures.

In fact, *d* values from the heat transfer approach represent the dynamic characteristic dimensions of the pore space among solid particles. Alternatively, the mean pore space among solid particles can be estimated from the porosity (*n*), dry bulk density (*ρ*_*b*_), and *SA* of a granular material^2^,


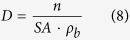


where *D* is the static mean separation of particles, i.e., the geometric mean dimension of the gas space among particles^2^.

## Materials and Methods

Two soils were used in our study. One soil, with a silty clay texture, had 7% sand, 50% silt, and 43% clay. The total soil specific surface area of the silty clay was measured using the ethylene glycol monoethyl ether (EGME) method following the procedure outlined by Pennell^5^. The second soil, with a sandy loam texture, had 45% sand, 50% silt, and 5% clay. The total soil specific surface area of the sandy loam was measured using the water adsorption method^6^. Soil samples were air dried, ground, and sieved through a 2-mm mesh. To determine thermal conductivity, oven-dried soil samples were packed into columns (70-mm inner diameter and 80-mm height) with a bulk density of 1.30 g cm^−3^ for the silty clay and 1.29 g cm^−3^ for the sandy loam. The experiment was performed in a temperature-regulated room (25 ± 1 °C).

For *λ* measurement under reduced air pressures, an apparatus similar to that adopted by Momose and Kasubuchi^7^ was used, with a minor difference that a single needle heat probe (Soiltronics, Burlington, WA, USA) was applied for determining *λ* under various air pressures. The probe had a diameter of 0.9 mm and a length of 60 mm. A vacuum pump was used to achieve a series air pressures from 101 kPa to vacuum. The heating power and soil temperature dynamics during the measurements were recorded with a datalogger (Model 23X, Campbell Scientific, Logan, UT, USA). For more details of the line-source heat probe method, please refer to Shiozawa and Campbell^8^.

For *λ* measurement under increased air pressures, the samples were placed in a pressure chamber connected to a compressor (Soil Moisture Equipment Corp., Santa Barbara, CA, USA). Four absolute pressures, 101, 202, 404, and 1010 kPa, were applied to the packed soil columns. Soil thermal conductivity measurement process was the same as that under reduced air pressure. More details about *λ* measurement process under increased air pressures could be found in Lu *et al*.^9^.

## Results and Discussion

**Figure 1:**
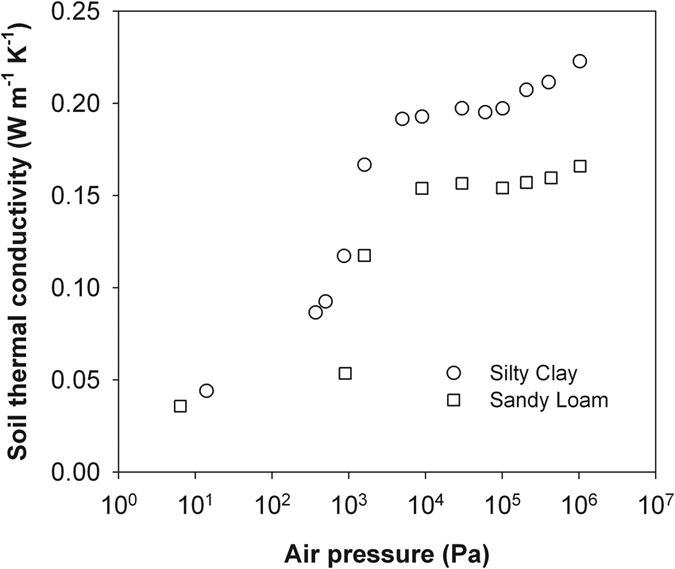
Dependence of thermal conductivity of the dry soils on air pressure.

Figure 1 presents the results of soil *λ* as a function of air pressure for the two soils of different textures in semi-logarithmic scale. At air pressures near vacuum, *λ* was the lowest because of the lowest air density of rarefied gas, and the conduction transport through the solid particles dominates soil heat transfer. A sharp *λ* rise was observed between 1000 Pa to 10000 Pa due to the contribution of increased air density in the pores to soil heat transfer. Masamune and Smith^10^ found similar phenomena and explained as the influence of gas enclosure among particles: the thermal conductivity of gas *λ*_g_ in a porous media is directly related to air pressure. At the air pressure larger than 10000 Pa, further increasing of *λ* might be from the increased contribution of micro-pores. A larger gas density and a lower mean free path promote more air molecules entering into the intra-aggregate pores, which contributes to the heat transfer finally.

**Figure 2:**
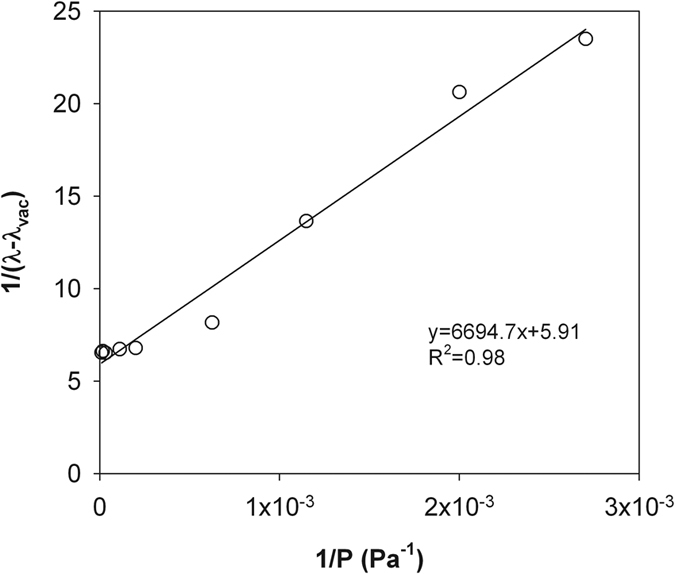
The measured 1/(*λ* − *λ*_vac_) and 1/P relationship for the dry silty clay under reduced air pressures. The solid line represents the linear regression result. Note: *λ*, soil thermal conductivity; *λ*_vac_, soil thermal conductivity under vacuum; P, air pressure.

**Figure 3:**
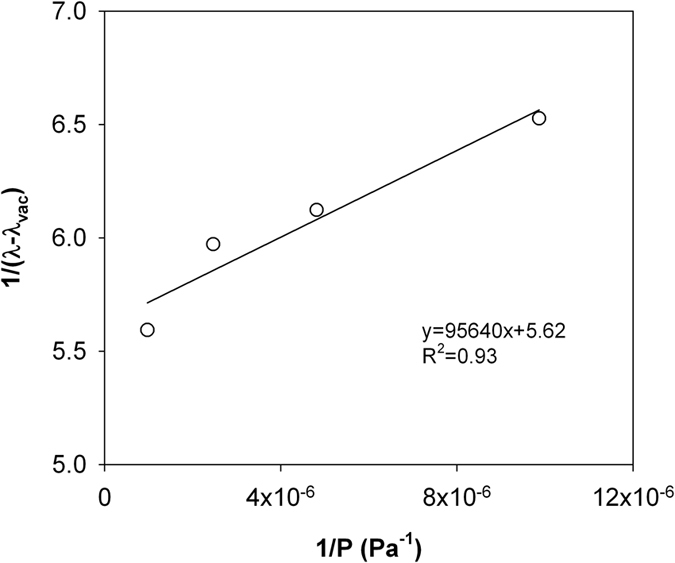
The measured 1/(*λ* − *λ*_vac_) and 1/P relationship for the dry silty clay under increased air pressures. The solid line represents linear regression result. Note: *λ*, soil thermal conductivity; *λ*_vac_, soil thermal conductivity under vacuum; P, air pressure.

**Figure 4:**
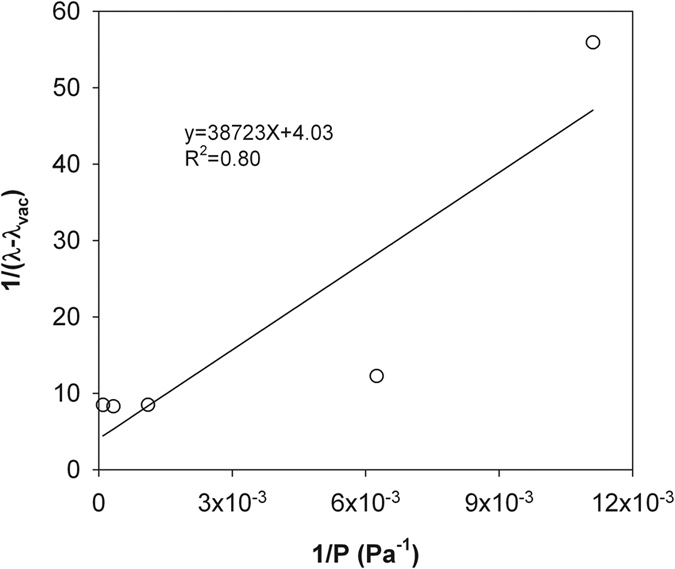
The measured 1/(*λ* − *λ*_vac_) and 1/P relationship for the dry sandy loam under reduced air pressures. The solid line represents the linear regression result. Note: *λ*, soil thermal conductivity; *λ*_vac_, soil thermal conductivity under vacuum; P, air pressure.

**Figure 5:**
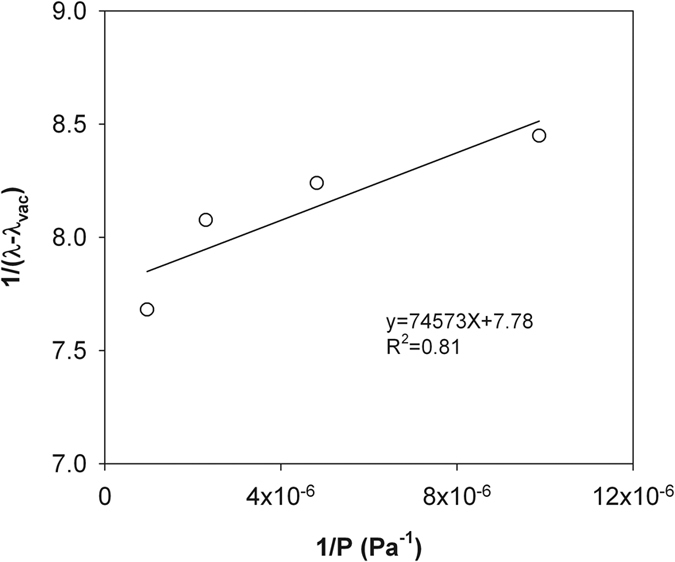
The measured 1/(*λ* − *λ*_vac_) and 1/P relationship for the dry sandy loam under increased air pressures. The solid line represents linear regression result. Note: *λ*, soil thermal conductivity; *λ*_vac_, soil thermal conductivity under vacuum; P, air pressure.

Similar to Momose and Kasubuchi^2^, we took the *λ* value at the lowest air pressure (i.e., 14 Pa in silty clay and 6.4 Pa in sandy loam) as *λ*_vac_ and investigated the changes of 1/(*λ* − *λ*_vac_) as a function of 1/*P* under reduced (<101 kPa) and increased (>101 kPa) air pressures (Figs 2, 3, 4 and 5). In both cases, strong linear relationships were observed between 1/(*λ *− *λ*_vac_) and 1/*λ*. However, for both soils, the slopes of the straight lines indicated that the rate of 1/(*λ* − *λ*_vac_) change under increased air pressure was much greater than that under reduced air pressure. Thus, the kinetic theory-based Eq. (7) is appropriate for describing the thermal conductivity and air pressure relationship, and can be applied for estimating *d* based on a series measurement of *λ* and *P*.

Under our experimental conditions, the estimated *d* values of the silty clay were 6.0 × 10^−3^ and 3.9 × 10^−4^ mm from reduced and increased air pressure data, respectively (Table 1), which were 2801 and 183 times larger than the *D* value obtained using the geometrical method (Table 1). For the sandy loam soil, the estimated *d* from reduced and increased air pressure methods were 7.1 × 10^−4^ and 6.9 × 10^−4^ mm, respectively, which were 51 and 50 times larger than the geometrical *D* value. Apparently the heat transfer-based method significantly overestimated *d*, but the increased air pressure approach produced better estimations than that of the reduced air pressure method. The overestimation by the heat transfer based method is caused by the fact that the soils (especially the silty clay) have larger surface areas, and conduction through solid particles is the dominant heat transfer mechanism. In the Momose and Kasubuchi^2^ study, the estimated *d* values under reduced air pressure were 200–300 times larger than the SA derived *D* value. These SA value, however, was obtained using the N_2_ adsorption method, which only considered the external surfaces of soil pores^5^ and ignored the surfaces area of numerous micro pores. Thus, we expect greater discrepancies between *d* and *D* occurs if the total SA was incorporated in the Momose and Kasubuchi^2^ study. To obtain accurate comparisons, the *D* values in this study were calculation from SA measurements that provided the total (external + internal) soil surface area^5^,^6^.

For the sandy loam soil (5% clay), the *d* values of under reduced air pressure was slightly larger than that under increased air pressures (Table 1). For the silty clay soil (43% clay), however, *d* estimates under reduced air pressure was 15.3 times that under increased air pressures. These results suggest that the difference of *d* estimates between the reduced and increased air pressures becomes larger with increasing soil clay content.

Another feature of Table 1 is that the *d* estimates from the heat transfer approach were not constant in the silty clay soil. This may be arisen from its large clay content (43%) and complicated interior pore structures that have various pores with dimensions vary from mm to nm^11^. Through the binding of soil organic materials, the majority of clay particles form aggregates, which create numerous complicated inter-aggregate and intra-aggregate micropores. This is better illustrated by the changes of mean free path *l* of air molecules as a function of air pressures (Table 2). In our study, *l* of the silty clay varied from 0.48 mm to 7 × 10^−6^ mm when air pressure was increased from 14 Pa to 1.03 MPa. This indicates that in most cases, collisions within gas molecules and between gas molecules and solid surface occur simultaneously. In the case of reduced air pressure, air molecules in inter-aggregate pores, rather than in intra-aggregate pores, contribute primarily to the decrease of thermal conductivity^2^. Under the situation of increased air pressure, a larger contribution from intra-aggregate pores to heat transfer might occur due to more collisions between gas molecules and solid surface in micro pores. In other words, the gas space length is smaller than *l* in one case and is lager than *l* in another case, and under the experimental air pressures, the two phenomena co-exist in the silty clay soil. Under reduced air pressures, few collisions occur in intra-aggregate pores due to the larger *l* and low air density. Thus, *d* estimates represent mainly the characteristics of inter-aggregate pores. Under the situation of increased air pressure with smaller *l* and high air density, micro-pores contribution to heat transfer becomes greater because of more collisions between gas molecules and solid surface in these pores. As a result, more micro-pore structures are revealed in *d* estimates.

Overall, it is reasonable to suggest that thermal conductivity data under increased air pressures reveals more information about soil micro-pore structures, and better estimation of characteristic dimension of the pore space than the measurements under reduced air pressures.

## Conclusions

Soil heat transfer features under various air pressures are related closely to separation of solid particles, which reflect the traits of soil micro-pore structures. In this study, we measured soil thermal conductivity in two-phase soil systems under reduced and increased air pressures, from which the characteristic pore space *d* was estimated. In both cases, *d* results were significantly larger than the corresponding *D* data, suggesting that conductive heat transfer through solid particle was the dominant mechanism in dry soils. Compared to the reduced air pressure method, the increased air pressure approach significantly improved the accuracy of characteristic dimensions of the pore spaces. Thus, soil thermal conductivity measurement under increased air pressure can help to illustrate heat transfer mechanism in micro-scale, and provide a better estimation of soil micro-pore structure than that with reduced air pressure.

## Additional Information

**How to cite this article:** Lu, S. *et al*. Thermal separation of soil particles from thermal conductivity measurement under various air pressures. *Sci. Rep.* **7**, 40181; doi: 10.1038/srep40181 (2017).

**Publisher's note:** Springer Nature remains neutral with regard to jurisdictional claims in published maps and institutional affiliations.

## Figures and Tables

**Table 1:** Mean separation of particles determined using the reduced air pressure (*d*_*1*_), increased air pressure (*d*_*2*_), and geometrical methods (*D*).

	Mean separation of particles (mm)	
	Silty clay	Sandy loam
Estimation from reduced air pressure method (*d*_*1*_)	6.0 × 10^−3^	7.1 × 10^−4^
Estimation from increased air pressure method (*d*_*2*_)	3.9 × 10^−4^	6.9 × 10^−4^
Geometrical mean separation of particles (*D*)	2.2 × 10^−6^	14 × 10^−6^

**Table 2:** Calculated mean free paths of air molecules (*l*) and the corresponding experimental air pressures (*P*).

*P* (Pa)	*l* (mm)
14	0.48
370	0.018
500	0.014
870	7.8 × 10^−3^
1600	4.2 × 10^−3^
5000	1.4 × 10^−3^
9000	7.5 × 10^−4^
30000	2.3 × 10^−4^
60000	1.1 × 10^−4^
101325	6.7 × 10^−5^
207716	3.3 × 10^−5^
405300	1.7 × 10^−5^
1028449	7.0 × 10^−6^
